# Increased in carbon isotope ratios of Brazilian fingernails are correlated with increased in socioeconomic status

**DOI:** 10.1038/s41538-020-0069-1

**Published:** 2020-07-16

**Authors:** Gabriela Bielefeld Nardoto, João Paulo Sena-Souza, Tiago Borges Kisaka, Fábio José Viana Costa, Paulo José Duarte-Neto, James Ehleringer, Luiz A. Martinelli

**Affiliations:** 1 Ecology Department, Institute of Biological Sciences, University of Brasília, Asa Norte, Brasília, CEP 70910-900 Brazil; 2 Department of Geosciences, Universidade Estadual de Montes Claros, Montes Claros, MG Brazil; 3 National Institute of Criminalistics, Federal Police, Asa Sul, Brasília, CEP 70610-200 Brazil; 4 Department of Statistics and Informatics, Rural Federal University of Pernambuco, R. Manuel de Medeiros, 35, Dois Irmãos, Recife, CEP 52171-050 Brazil; 5 School of Biological Sciences, University of Utah, 257 South 1400 East, Salt Lake City, UT USA; 6 Laboratory of Isotope Ecology, Center for Nuclear Energy in Agriculture, University of São Paulo, Av. Centenário, 303, São Dimas, Piracicaba, CEP 13416-000 SP Brazil

**Keywords:** Environmental social sciences, Health sciences

## Abstract

High *δ*^13^C in human tissues in Brazil indicate high consumption of C_4_-based sources due to the consumption of highly processed food and animal protein. The significant positive correlation between the human developed index (HDI) developed by the United Nations Development Program, and fingernail *δ*^13^C at the county level proved to be useful as a new proxy in tracking human nutrition. Regions with higher HDI are those with higher consumption of highly processed food.

## Introduction

Several studies have used the multi-isotope approach to track the geographic origin of human remains as well as human movement in forensic anthropology^1^, but only a few studies have used it in association with socioeconomic status and dietary behavior changes^2^. These studies have demonstrated the progressive substitution of local food staples by industrialized processed foods in the developing regions of the world (the so-called nutrition transition process)^3^. In this context, we explored stable isotopic analysis as a new proxy for human nutrition status across Brazil under the worldwide “global supermarket” dietary trend. Brazil is a middle-income country with contrasting inequitable geographic regions in terms of socioeconomic development, health, and educational services^4^. We compared the carbon isotopic ratio of fingernails from residents of more economic developed regions of the country, where high consumption of processed food prevails, with the less developed regions, where locally produced food predominates, particularly staples like rice, beans, and cassava.

While C_3_ plants have *δ*^13^C varying approximately from −34 to −24‰, C_4_ plants have *δ*^13^C that varies from −14 to −10‰, with no overlap between these plant types^5^. Therefore, in recent decades, carbon isotopic composition became a useful tool to track C_3_ and C_4_ carbon in biological systems worldwide^6^. Despite the fact that pineapple that is a CAM plant that could confound a straightforward interpretation of C_3_ vs. C_4_ diet contribution^5^. its consumption has no significant impact in the Brazilian diet. In fact, fruits and vegetables provide approximately only 2% of total daily calories to Brazilians^6^.

Brazil is mostly a tropical country with proper climatic conditions to grow C_4_ crops; among them, C_4_ pastures cover an area of almost 200 million ha^4^. Although soy occupies almost 35 million ha, corn is the second arable crop in the country covering almost 20 million ha, and sugarcane covering 10 million ha is the third. Due to these vast productive areas, C_4_ carbon sources in Brazil are generally an inexpensive alternative for the food industry either as sugar or high-fructose corn syrup. Accordingly, C_4_ carbon is available in the country in several energy-dense processed foods like dairy, various types of processed meats, beverages (soft drinks, wine, and beer), cakes, crackers, and even unexpectedly, in products like soy sauce^7,8^.

This in part explains why Brazilians residing in urban areas have higher *δ*^13^C in human tissues than residents of other countries^9,10^. However, in more isolated towns and villages in different geographic regions of Brazil where the degree of market integration is highly variable, the *δ*^13^C of human tissues reflects a diet based more on C_3_ plants (rice, beans, cassava), coupled with consumption of locally derived animal protein, such as fish or bushmeat, depending on the region^11^.

Motivated by the fact that Vale et al.^12^ reported a positive correlation between Human Development Index (HDI) and body weight at the state level in Brazil, our initial hypothesis was that at the county level, the HDI and fingernail *δ*^13^C representing county residents [*δ*^13^C]_*m*_ would be positively correlated. In order to test this hypothesis, we correlated the HDI at the county level [HDI]_*m*_ with the [*δ*^13^C]_*m*_ (see “Methods” for definition) among 37 counties distributed in different geographic regions of Brazil (Fig. S1—Supplementary material).

## Results and discussion

**Fig. 1:**
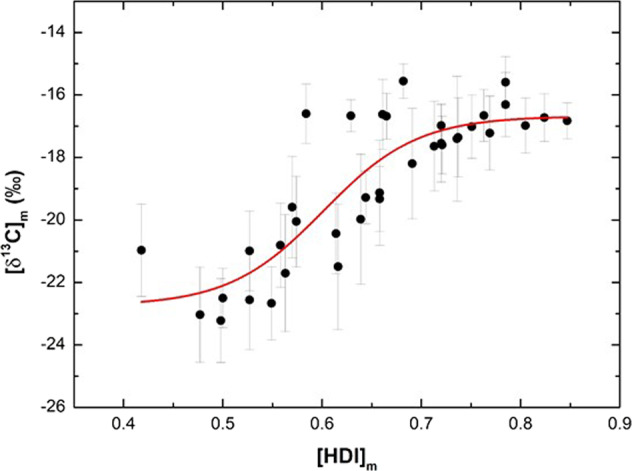
Human Development Index at county-level vs. average *δ*^13^C of fingernails. Bi-plot of county-level human development index ([HDI]_*m*_) for 37 counties (see “Methods”) and average *δ*^13^C of fingernails obtained as an average for residents of each municipality (*δ*^13^C_*m*_). Bars represent the standard deviation and the red line, the Boltzmann’s sigmoidal line.

We found a strong positive sigmoidal correlation (Fig. 1) between these two parameters according to the Boltzmann’s equation (*R*^2^_Adj._ = 0.72, Reduced *χ*^2^ = 1.55 by Levemberg–Marquadt algorithm):

1 $$\delta ^{13}\mathrm{C} = \left( {\frac{{ - 22.75\left( { \pm 0.53} \right) + 16.68}}{{1 + e^{\frac{{\mathrm{HDI} - 060( \pm 0.024)}}{{0.048( \pm 0.021)}}}}}} \right) - 16.68( \pm 1.07).$$

In this sense, Eq. (1) suggests higher consumption of processed food in areas with higher HDI, where market integration and purchasing power are higher than less developed areas of the country. This finding is in line with Vale et al.^12^, confirming our initial hypothesis.

**Fig. 2:**
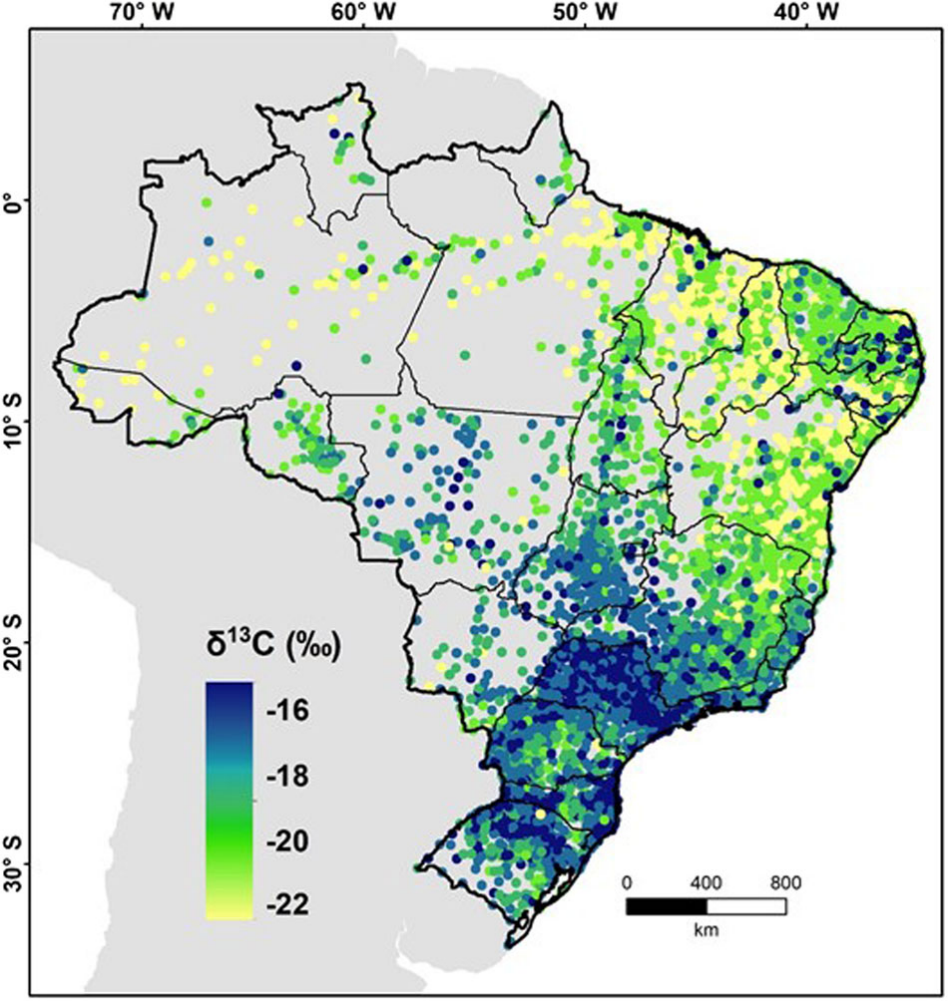
Average *δ*^13^C for each municipality estimated by Eq. (1). Values of the average *δ*^13^C for each municipality ([*δ*^13^C]_*m*_) obtained through the correlation average *δ*^13^C of fingernails and [HDI]_*m*_ of 37 Brazilian counties (Eq. (1)). Each dot represents one municipality of Brazil using municipality centroid geographic coordinates.

Using the Eq. (1), the [*δ*^13^C]_*m*_ was estimated for the 5507 Brazilian counties (see “Methods”), and with these values a map of [*δ*^13^C]_*m*_ was generated in order to shed light on spatial trends for the country (Fig. 2). Although we found a robust correlation, future investigations on fingernails *δ*^13^C in municipalities with contrasting [HDI]_*m*_ and in different regions of Brazil should be conducted to validate our model. We see a clear spatial polarization between north and south with lower [*δ*^13^C]_*m*_ values in the north than in the south, which means that in the north, C_3_-like foods predominate in the diet, resembling Brazilian staple foods, while in the south, C_4_-like foods predominate, which represents a higher market integration and consumption of processed food (Fig. 2).

Therefore, if we accept that [*δ*^13^C]_*m*_ is a proxy for higher consumption of processed food and adherence to the supermarket diet, it seems that in the southern region, the so-called nutrition transition has already been completed, whereas in the northern region this transition is still taking place (Fig. 2).

If the [HDI]_*m*_ continues to grow, especially in counties of the northern states of the country, we will see an increase in [*δ*^13^C]_*m*_, indicating the late stage of the nutrition transition and a predominance of the supermarket diet with a high degree of market integration. Future trends of [*δ*^13^C]_*m*_ in the southern states of the country are more difficult to predict, if this part of the country adopts a plant-based diet that includes lower consumption of meats coupled with higher consumption of whole grain cereals, legumes, and fruits, we predict a decrease in the [*δ*^13^C]*m* of this region in the future.

The discussion above acknowledges that our model has potentially a limited time span according to the progress of the socioeconomic conditions of the country as a whole and its different regions. Our model also has a geographic-limited scope being only potentially valid in those countries where there are inexpensive sources of C_4_ dietary carbon, which is the case in most northern-temperate countries.

However, we believe that in countries like Brazil, the [*δ*^13^C]*m* could be another tool for detecting macro-scale trends in nutrition in time and space, since as emphasized by Walls et al.^13^, it is not easy to evaluate nutrition trends in low and medium-income countries, and the isotopic approach shown here could be also useful in linking social-economic dimensions to dietary trends. In addition, it provides new perspectives in human forensic anthropology as a powerful tool for tracking human movement in the contemporary world under the “supermarket diet” trend.

## Methods

The carbon isotope ratio (^13^C:^12^C) was determined in fingernails of residents of 37 Brazilian counties totaling almost 4500 samples (Supplementary Table 1; Supplementary Fig. 1). Briefly, fingernails were collected from donors using fingernail clippers. Samples were then cleaned with a solution of distilled water, methanol, and chloroform. For this survey, an authorization by the official Brazilian human ethical committee was previously submitted, approved, and received the registration number of COET 053, Piracicaba, São Paulo, Brazil.

The carbon isotopic ratio (^13^C:^12^C) in these samples was determined through a Delta Plus mass spectrometer for isotopic ratios (ThermoFisher Scientific), in the Laboratory of Isotope Ecology, CENA (University of São Paulo), Brazil. The results were reported as the deviation (*δ*) in parts per thousand (‰) relative to standard international references; *δX* = (*R*_sample_/*R*_std_ − 1) × 1000; where, *X* is carbon, *R* is the heavy to light isotope ratio for carbon (^13^C:^12^C) of the sample (*R*_sample_), and of the standard (*R*_std_), VPDB-Vienna Pee Dee Belemnite. BBOT (Fisons Instruments [C_26_H_26_N_2_O_2_S]) and “grounded leaves of sugarcane” were used as internal standards to calibration during analysis runs. Every ten runs, both internal standards were used as target sample. Long-term standard deviations of internal standards used at the Ecology Isotope laboratory are of 0.2‰ for carbon.

The fingernail *δ*^13^C aggregated at the municipality level ([*δ*^13^C]_*m*_) was obtained by averaging the *δ*^13^C of all donors from a municipality.

The counties included in this study represent 0.7% of the 5,507 Brazilian counties, and ~10% of Brazil’s population (Supplementary Table 1). These counties were chosen due to opportunities created by several scientific projects that resulted in a series of publications^9,7^. Details on sampling can be found in these publications. Not all samples were obtained in the same year, and this fact could be a limitation resulting in biased findings if temporal changes were large. However, most of the samples (almost 70%) were obtained from 2008 to 2015; 23% from 2002 to 2006; and 10% after 2015. Another important source of variability could be the fact that in some regions only urban centers were sampled, whereas in others, mainly in the Amazon region, small isolated villages were also included. This is important to mention because we have shown in other publications that urban centers are fully market integrated, and more isolated villages are not; these villages tend to have lower *δ*^13^C than urban centers in the same county^7^.

The HDI was created in 1990 by the United Nations as a response to persistent criticism throughout the 1980s that economic development alone could not capture human development (http://hdr.undp.org/en/content/human-development-index-hdi). Brazil was the first country to launch HDI at the municipality level ([HDI]_*m*_) using basically the same parameters of the HDI developed by the United Nations. We obtained HDI at the municipality level [HDI]_*m*_ for 2010, the last available year for Brazilian counties (http://www.atlasbrasil.org.br).

Optimal parameters of the Boltzmann’s sigmoidal model were obtained using a Levemberg–Marquadt algorithm of the Origin software (ver 8.6, Originlab). The Boltzmann sigmoidal equation used in the present work was:

2 $$y\left( x \right) = \left( {\frac{{A_1 - A_2}}{{1 + e^{\frac{{x - x_0}}{{\partial x}}}}}} \right) + A_2,$$

where *A*_1_ and *A*_2_ are the equilibrium values of the dependent variable before and after the transition, respectively; *x*_0_ is the inflection point and ∂x is the slope of the curve that describes the behavior of the process during the transition. The fingernail *δ*^13^C values of each Brazilian municipality was obtained based on predictions of [*δ*^13^C]_*m*_ from [ΗDΙ]_*m*_ according to the above equation, using municipality centroid geographic coordinates.

High consumption of seafood, with *δ*^13^C values close to C_4_ crops, could show a false positive correlation between *δ*^13^C of human tissues and consumption of meats and processed food. However, due to its high price, seafood consumption in Brazil is rather low, even in coastal cities as well as in marine fishing villages.^14^

## Supplementary information

Supplemental Material

## Data Availability

The data used in this article is in the Table 1 of the Supplementary material.
