# Supplementary material for: Increased in carbon isotope ratios of Brazilian fingernails are correlated with increased in socioeconomic status
Source: NPJ Sci Food. 2020 Jul 16;4:9. doi: 10.1038/s41538-020-0069-1 (PMC7366703; doi:10.1038/s41538-020-0069-1)
Supplement: Supplementary file 1 — Supplemental Material [file 41538_2020_69_MOESM1_ESM.docx]

**Supplementary Material**

Supplementary Table 1. Brazilian counties included in this study with longitude (Long) and latitude (Lat), population (Pop.), [δ^13^C]*_m_*, followed by standard deviation (sd), minimum (min) and maximum δ^13^C values, and number of samples (*n*) analyzed in each municipality.

| Municipality | Lat | Long | Pop | [δ^13^C]*_m_* | sd | max | min | *n* |
| --- | --- | --- | --- | --- | --- | --- | --- | --- |
|  |  |  | *x*1000 | (‰) | (‰) | (‰) | (‰) |  |
| Alto Paraíso de Goiás | -47.5191 | -14.1362 | 7.3 | -17.6 | 1.4 | -21.2 | -14.3 | 214 |
| Alvarães | -64.8135 | -3.2166 | 15.9 | -21.0 | 1.3 | -23.5 | -17.8 | 109 |
| Barcelos | -62.9257 | -0.9728 | 25.7 | -22.5 | 0.9 | -24.4 | -19.7 | 98 |
| Benjamin Constant | -70.0273 | -4.3750 | 42.0 | -20.1 | 1.5 | -22.9 | -17.1 | 92 |
| Brasília | -47.8879 | -15.7941 | 2974.0 | -16.7 | 0.8 | -18.8 | -14.7 | 65 |
| Cananéia | -47.9285 | -25.0169 | 12.2 | -17.5 | 1.2 | -19.0 | -14.8 | 8 |
| Carauari | -66.8966 | -4.8817 | 25.8 | -22.7 | 1.2 | -24.0 | -20.0 | 29 |
| Cavalcante | -47.4634 | -13.7993 | 9.6 | -16.6 | 1.0 | -18.2 | -13.9 | 38 |
| Colinas do Sul | -48.0768 | -14.1502 | 3.4 | -19.1 | 1.7 | -23.4 | -15.7 | 40 |
| Cosmópolis | -47.1968 | -22.6458 | 58.8 | -17.2 | 1.2 | -20.7 | -15.2 | 32 |
| Cuiabá | -56.0949 | -15.5989 | 551.0 | -15.6 | 0.8 | -17.5 | -14.4 | 25 |
| Eirunepe | -69.8663 | -6.6674 | 30.7 | -21.7 | 1.9 | -25.0 | -17.7 | 39 |
| Florianópolis | -48.5476 | -27.5878 | 421.4 | -16.8 | 0.6 | -18.3 | -15.6 | 67 |
| Guamaré | -36.3181 | -5.1074 | 15.3 | -16.7 | 0.5 | -17.6 | -15.7 | 30 |
| Itacotiara | -58.4402 | -3.1404 | 100.0 | -19.3 | 0.8 | -21.0 | -17.4 | 34 |
| Itamarati | -68.2479 | -6.4378 | 8.0 | -23.0 | 1.5 | -25.2 | -17.4 | 81 |
| Macau | -36.6350 | -5.1134 | 31.6 | -16.7 | 0.7 | -19.9 | -15.0 | 161 |
| Maceió | -35.7016 | -9.6608 | 932.7 | -17.6 | 0.9 | -21.7 | -15.2 | 110 |
| Manacapuru | -60.6206 | -3.2983 | 96.2 | -20.4 | 1.3 | -23.8 | -18.1 | 149 |
| Manaus | -60.0233 | -3.1347 | 2145.5 | -17.4 | 1.3 | -21.2 | -14.6 | 111 |
| Maraã | -65.5743 | -1.8664 | 18.2 | -23.2 | 1.3 | -26.9 | -19.2 | 279 |
| Melgaço | -50.7175 | -1.8034 | 24.8 | -21.0 | 1.5 | -22.9 | -17.9 | 36 |
| Mossoró | -37.3569 | -5.1938 | 237.2 | -17.0 | 0.7 | -19.0 | -14.8 | 220 |
| Natal | -35.2523 | -5.7509 | 803.7 | -16.7 | 0.8 | -19.7 | -14.9 | 111 |
| Nova Olinda | -59.0931 | -3.8876 | 35.9 | -20.8 | 1.3 | -24.6 | -18.2 | 138 |
| Novo Airão | -60.9488 | -2.6234 | 18.9 | -19.6 | 1.6 | -22.5 | -16.6 | 23 |
| Ouro Preto do Oeste | -62.2611 | -10.7236 | 37.9 | -15.6 | 0.5 | -16.6 | -14.7 | 10 |
| Parintins | -56.7363 | -2.6271 | 102.0 | -19.3 | 1.0 | -21.8 | -17.5 | 32 |
| Piracicaba | -47.6468 | -22.7237 | 364.6 | -16.3 | 1.0 | -20.4 | -12.2 | 387 |
| Santarém | -54.7186 | -2.4362 | 297.6 | -18.2 | 1.8 | -22.5 | -15.1 | 93 |
| São Paulo | -46.5704 | -23.5674 | 11253.5 | -17.0 | 0.9 | -20.2 | -13.5 | 911 |
| Tabatinga | -69.9462 | -4.2319 | 62.3 | -21.5 | 2.0 | -24.9 | -17.8 | 96 |
| Tefé | -64.7084 | -3.3484 | 62.6 | -20.0 | 2.1 | -24.5 | -15.1 | 179 |
| Teresina de Goías | -49.0918 | -16.4790 | 3.0 | -16.6 | 1.1 | -19.6 | -14.8 | 27 |
| Uarini | -65.1553 | -2.9836 | 13.3 | -22.6 | 1.6 | -26.1 | -17.3 | 279 |
| Ubatuba | -45.0721 | -23.4360 | 78.8 | -17.0 | 1.0 | -19.3 | -13.4 | 120 |


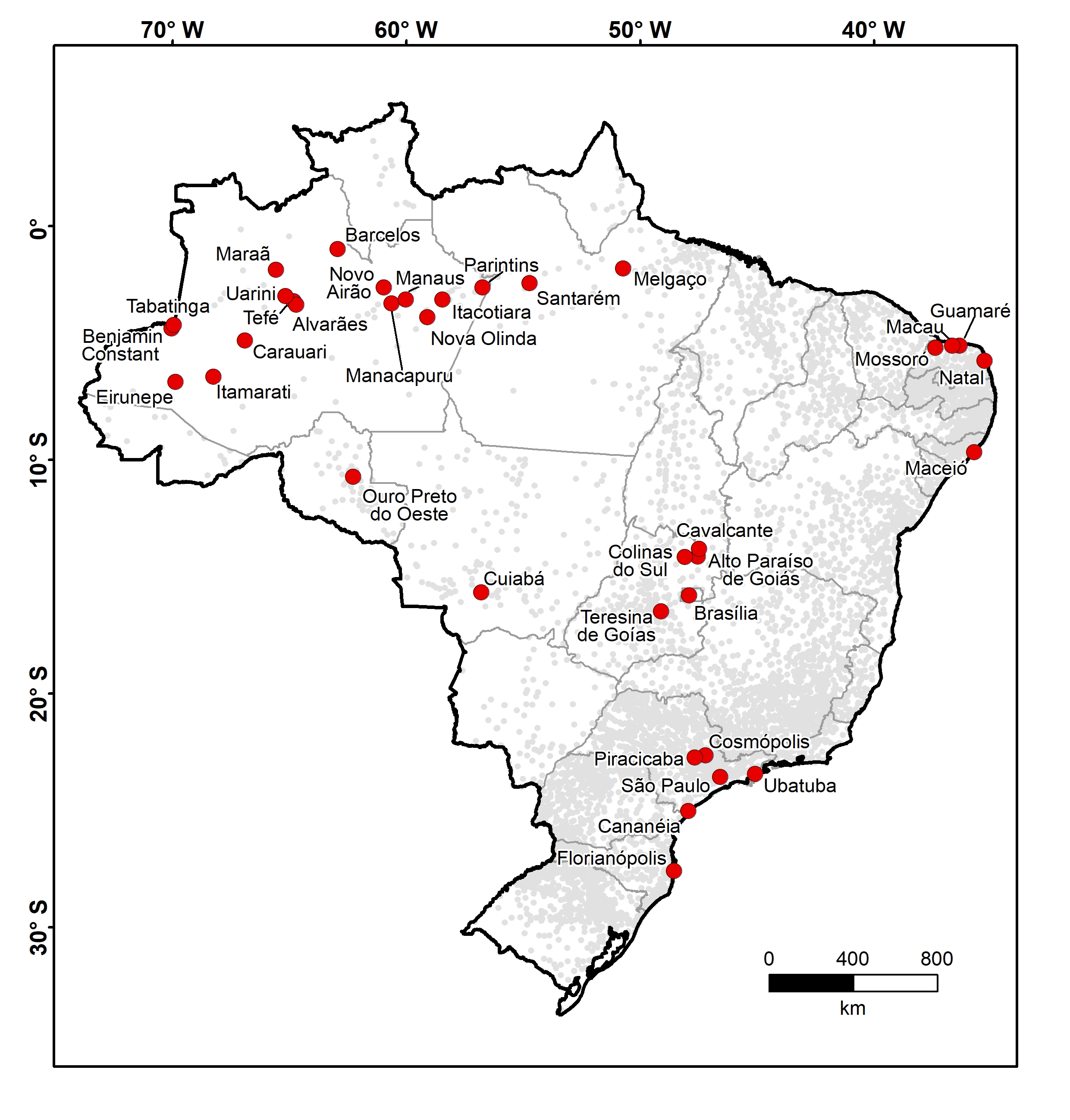


Supplementary Figure 1. Map of Brazil with States border (grey lines) and municipalities (grey dots). Municipalities represented by red dots are those with fingernails isotopes data listed in Table 1 of the Supplementary Material.
